# Fetal *de novo* mutations and preterm birth

**DOI:** 10.1371/journal.pgen.1006689

**Published:** 2017-04-07

**Authors:** Jingjing Li, John Oehlert, Michael Snyder, David K. Stevenson, Gary M. Shaw

**Affiliations:** 1 Division of Neonatal and Developmental Medicine, Department of Pediatrics, Stanford University, School of Medicine, Stanford, CA, California, United States of America; 2 Department of Genetics, Center for Genomics and Personalized Medicine Stanford University, School of Medicine, Stanford, CA, California, United States of America; Case Western Reserve University School of Medicine, UNITED STATES

## Abstract

Preterm birth (PTB) affects ~12% of pregnancies in the US. Despite its high mortality and morbidity, the molecular etiology underlying PTB has been unclear. Numerous studies have been devoted to identifying genetic factors in maternal and fetal genomes, but so far few genomic loci have been associated with PTB. By analyzing whole-genome sequencing data from 816 trio families, for the first time, we observed the role of fetal *de novo* mutations in PTB. We observed a significant increase in *de novo* mutation burden in PTB fetal genomes. Our genomic analyses further revealed that affected genes by PTB *de novo* mutations were dosage sensitive, intolerant to genomic deletions, and their mouse orthologs were likely developmentally essential. These genes were significantly involved in early fetal brain development, which was further supported by our analysis of copy number variants identified from an independent PTB cohort. Our study indicates a new mechanism in PTB occurrence independently contributed from fetal genomes, and thus opens a new avenue for future PTB research.

## Author summary

Preterm birth is a prevalent pregnancy condition and leads to substantial morbidity and mortality. Its genetic association has been well observed, but the underlying etiology remains unclear. Current research has been focused on identifying risk factors in maternal genomes. In this study, we tested an unexplored hypothesis that preterm birth could be independently influenced by fetal genomes. We analyzed fetal *de novo* mutations, those not inherited from parents, from 816 trio families, and found preterm infants tended to have increased de novo mutation rates compared to infants born at term. Importantly, we also observed that these preterm-associated *de novo* mutations preferentially affect dosage sensitive genes that are essential in embryonic development, and these affected genes are involved in early fetal brain development. Overall, our study for the first time showed the fetal genetic contribution to preterm birth, and suggested abnormal fetal development as a potential cause for preterm birth.

## Introduction

Preterm birth (PTB, delivery at less than 37 weeks of gestation) affects ~10–12% of newborns in the US[1, 2], and is the leading cause for neonatal morbidity and mortality[3, 4]. In addition to environmental factors, e.g. smoking during pregnancy[5, 6], there is evidence for a genetic component in PTB etiology. The heritability of PTB was estimated to be 25%-40% in a Swedish population[7], 17%-27% in an Australian population[8], and 13.3%-24.5 in the Utah population[9]. Epidemiological studies reveal that PTB is associated with familial PTB histories[10–12], and indicate that the genetic component in PTB should be largely explained by the “maternal inheritance”, but not significantly from paternal genetics[13, 14]. Therefore, genetic association studies or candidate gene analysis have been performed on maternal and/or fetal genomes, and identified genes in infection, inflammation, and innate immunity that likely predispose pregnancies to PTB[15–17].

In this study we directly tested an unexplored hypothesis, where fetal *de novo* mutations, those not inherited from parents, increase PTB risk. This hypothesis, therefore, seeks to describe a genetic mechanism for PTB *solely* from fetal genomes. This possibility has been suggested by several studies: (1) elevated PTB frequency has been observed among fetuses with certain genetic disorders (e.g. the Ehlers-Danlos syndrome) even when the mothers are unaffected[18]. Therefore, it is likely that PTB is associated with genetic disorders that are caused by *de novo* mutations in fetal genomes. (2) If fetal *de novo* mutations indeed play a role, paternal age would be anticipated to exert an effect on PTB risk because it is well known that the *de novo* mutation rate is positively correlated with increasing paternal age[19–21]. Interestingly, by controlling for maternal age, analysis of more than 70,000 singleton births revealed an association between PTB risk and paternal age, where paternal age greater than 50 years old was associated with an odds ratio of 2.1 for PTB risk[22]. Studying *de novo* mutations is fundamentally different from previous genetic studies of PTB such as genome-wide association studies, including those targeting fetal genomes. This is because the role of *de novo* mutations implies a novel etiologic contribution to PTB *solely* from fetal genomes, in contrast with the association studies for common and inherited genetic mutations from parental genomes. Further, like many other complex human diseases, genome-wide association studies thus far have not yet identified robust signals for at-risk loci for PTB, which motivated us to study PTB from other complementary etiologic perspectives.

In this study, by analyzing high-coverage whole genome sequencing data from 816 parent–offspring trio families, we directly tested the hypothesis that PTB occurrence was associated with *de novo* mutations in fetal genomes. Our study revealed a significantly increased *de novo* mutation burden in PTBs relative to births at term. Analyzing genes affected by *de novo* mutations, we found that these genes were dosage sensitive, developmentally essential, and were significantly involved in early fetal brain development, suggesting a new mechanism in PTB due to fetal developmental abnormalities. For the first time, our study has identified a role of fetal *de novo* mutations in PTB etiology, and associated fetal brain developmental programming with PTB, thus offering critically important new biologic avenues for future PTB research.

## Results

**Fig 1:**
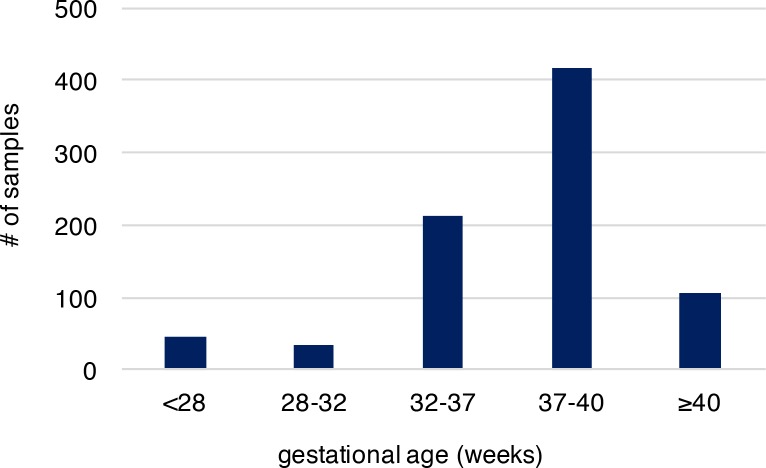
The gestational age distribution of the study participants. Preterm birth is considered if gestational age is less than 37 weeks. The cohort includes 292 preterm newborns and 524 newborns at term. Gestational age is by weeks.

We analyzed recently published whole-genome sequencing (WGS) data (with coverage ~60X), where *de novo* mutations were identified from whole blood samples of 816 parent-offspring trios to reveal associated genomic features[20]. Parents in these trios described are in good general health, not of high body mass index, without history of drug and alcohol abuse during pregnancy, nor exposure to hazardous chemicals. All members of these trios including the infants had no known chromosomal abnormalities, genetic diseases and specifically the infants had no gross structural anatomic anomalies[20]. Provision of gestational age (personal communication with Dr. Wendy Wong) associated with these published sequence data allowed us to explore the molecular basis of PTB, which was not systematically examined in the original study. Overall, 36,441 autosomal *de novo* mutations were identified in infants of the 816 trio families (only one sibling was studied when a family had a monozygotic twin pair), including 35,793 single nucleotide variants (SNVs) and 648 small insertions/deletions (indels). The high-quality of these *de novo* mutations was established in the original study[20]. Among the infants of the 816 trios, 292 were PTB (gestational age < 37 weeks, S1 Table), and the remaining 524 were term births (gestational age ≥ 37 weeks, S1 Table). Distribution of gestational age of PTB and term births is shown in **Fig 1,** where the mean gestational age of PTB and the non-PTB is 33.1 and 39.1 weeks, respectively, with the minimum of 22.7 weeks (S2 Table). We posited that if the occurrence of *de novo* mutations in fetal genomes is a significant contributor to PTB, two predictions follow: (1) PTB infants are expected to have increased *de novo* mutation burden relative to term infants; (2) the affected genes are significantly involved in prenatal, rather than postnatal, development. We tested these two predictions.

### Increased de novo mutation burden in PTB fetal genomes

**Fig 2:**
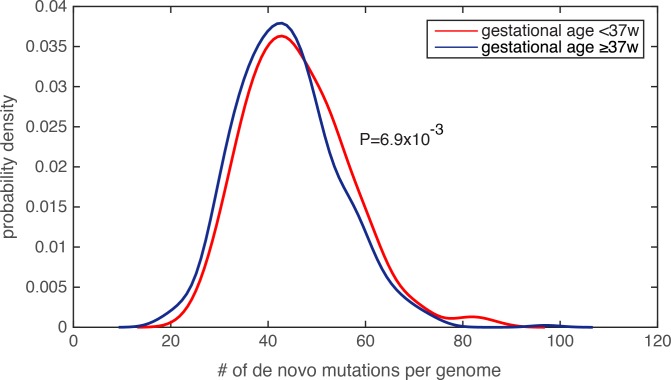
Significantly increased de novo mutation burden in preterm newborn’s genomes. The distribution of the number of de novo mutations per genome was compared between the PTB and non-PTB cohorts (P = 6.9e-3), and statistical significance was determined by Wilcoxon rank-sum test. Kernel density estimation was used to derive the probability density functions.

Because the amount of *de novo* mutations in personal genomes is strongly scaled by paternal age[19–21], and is modestly (or weakly) correlated with maternal age[20, 23], we first examined the parental age distribution, and found that the paternal and maternal age distributions were similar between the PTB and term infants (paternal age for PTB was 33.9±6.1 and for non-PTB was 33.5±5.8, P = 0.25, Wilcoxon rank-sum test; maternal age for PTB was 31.7±5.1 and for non-PTB was 31.4±4.9, P = 0.31, Wilcoxon rank-sum test, S2 Table). Finding insufficient evidence that parental ages were potential confounders, we compared the number of *de novo* mutations in each infant genome, and observed a significant increase in the *de novo* mutation burden in PTB infants relative to term infants (**Fig 2,** P = 6.9e-3, Wilcoxon rank-sum test, S3 Table). Notably, by identifying individuals with extreme *de novo* mutation load (the top 5% across all 816 subjects), we did not observe a statistical difference in paternal age between PTB and term groups (P = 0.62, Wilcoxon rank-sum test), nor in maternal age (P = 0.53, Wilcoxon rank-sum test). We performed two additional tests to ensure that the increased *de novo* mutation load in PTB cases was not resultant from unequal parental age distribution in this group. First, we performed logistic regression to combinatorially model paternal age, maternal age and the number of *de novo* mutations in each infant genome, which served to assess their individual effects on predicting the binary preterm status (as the response variable in the logistic model, Methods and Materials). Only the regression term for *de novo* mutation load exhibited a significant statistical association with preterm status (regression coefficient was 0.27, P = 4.1e-3), and the terms for parental ages did not (P>0.5, S3 Table). Second, we observed that Pearson’s correlation between paternal age and *de novo* mutation load across the 816 trios was 0.62, suggesting that ~38% (R^2^) of the variability in *de novo* mutation load could be explained by paternal age differences. Therefore, we fit the *de novo* mutation counts (the response variable) with the paternal ages (the explanatory variable) across the 816 family trios, and only considered the residuals of the *de novo* mutation count after subtracting the effect from paternal age. Again, the corrected *de novo* mutation counts (the residuals) consistently exhibited a significant increase in the PTB group relative to the term group (P = 6.3e-3. Wilcoxon rank-sum test). Similar analysis was also performed on maternal age, and confirmed the same observation (P = 8.7e-3, Wilcoxon rank-sum test).

Lastly, because generation of *de novo* mutations is strongly driven by DNA replication timing[19], we compared mean DNA replication timing[24] between the PTB and the term infant groups over a 1kb sequence window centered at each *de novo* mutation (Methods and Materials), and again found no statistically significant difference between the two groups (P = 0.23, Wilcoxon rank-sum test). Taking together, we observed significantly increased *de novo* mutation burden in PTB genomes, which is unlikely explained by uneven distributions of maternal age, paternal age, or DNA replication timing. Specifically, referenced with the average of 43.86 *de novo* mutations per term infant genome, the average increased to 46.08 per PTB genome, a significant figure considering the rate of 1e-8 *de novo* mutations per generation[21].

### Genes affected by PTB de novo mutations are developmentally essential

To understand potential functional consequences, we analyzed the *de novo* mutations affecting protein coding sequences (Methods and Materials), and identified 169 and 339 non-synonymous *de novo* mutations in PTB and term newborns (S4 Table), respectively, including missense, nonsense and frameshift mutations. We further annotated nonsynonymous mutations using the CADD (Combined Annotation Dependent Depletion) algorithm, which has the highest accuracy in identifying pathogenic and deleterious amino-acid changing substitutions[25]. In total, we identified 51 and 112 potentially consequential *de novo* mutations (i.e. deleterious missense, nonsense and frameshift mutations, see Methods and Materials) affecting 51 and 111 protein-coding genes (by RefSeq annotation) in PTB and term groups, respectively (S4 Table). Individuals (both PTB and term birth) carrying these identified deleterious mutations followed similar gestational age distribution as shown in Fig 1.

**Fig 3:**
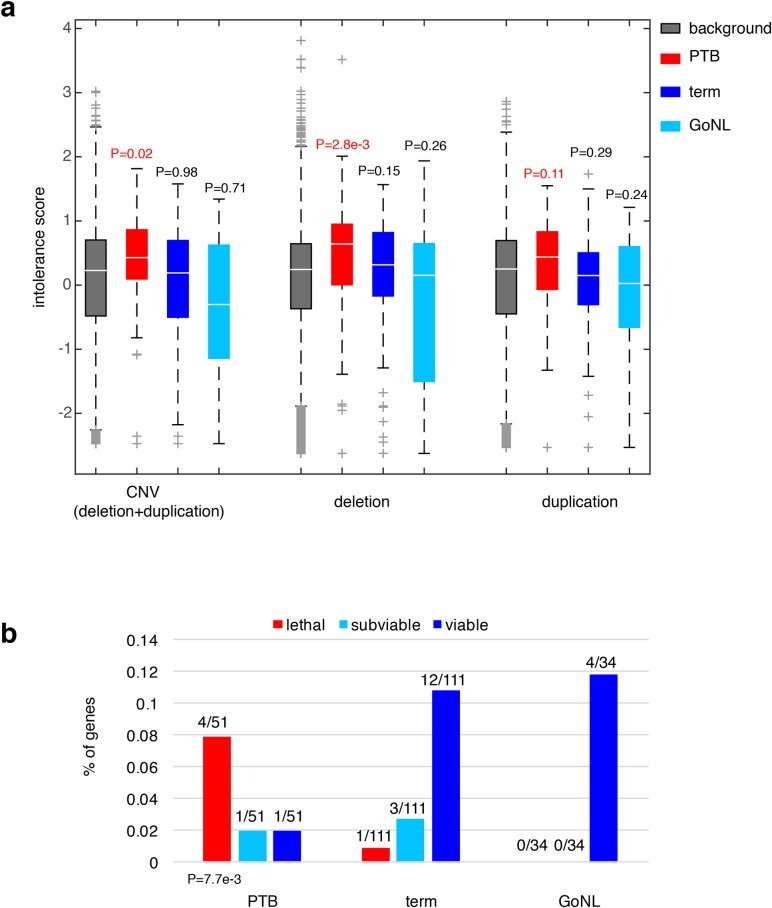
Characterization of the PTB genes. **(a)** Referenced with the genome background, the PTB gene set displayed a significant increase in their intolerance to copy number variations, particularly pronounced to deletion events, but not to duplication events. The same pattern was absent from the term gene set as well as from the GoNL genes. Referenced with the genome background, P values were calculated based on the Wilcoxon rank-sum test. **(b)** The percentages of lethal, subviable and viable genes in PTB, term and GoNL gene sets. The PTB set was significantly enriched for genes whose mouse orthologs are embryonic essential (i.e. homozygous knockouts displayed embryonic lethal phenotypes), whereas the term and GoNL genes had more viable genes (i.e. homozygous knockouts are viable). The P value was computed using the Fisher’s exact test by comparing the proportion of essential and viable genes in the PTB and term sets. Note that the GoNL set had no lethal and subviable genes.

We sought to understand the consequences of ablating these affected genes in PTB. We first considered their dosage effects based on a recent study, where gene intolerance to copy number variation (CNV) was quantified from a cohort of ~60,000 human exomes[26]. Previous studies have shown that essential or haploinsufficient genes are intolerant to CNVs, whereas genes in recessive disorders are more tolerant[26]. For the 51 genes we observed to be adversely affected by PTB *de novo* mutations, their CNV intolerance showed a substantial elevation from the genome background (P = 0.02, Wilcoxon rank-sum test, **Fig 3A**); however, the increase was not observed among the 111 genes identified from the term group of infants (P = 0.98, Wilcoxon rank-sum test, **Fig 3A**). For further confirmation, we analyzed the published GoNL control cohort[19], where *de novo* mutations were identified by whole-genome sequencing of 250 Dutch parent-offspring families (with no known diseases). Applying the same procedure described above, we identified 34 genes affected by deleterious GoNL *de novo* mutations. Again, this set of GoNL genes showed no increase in CNV intolerance (P = 0.71, Wilcoxon rank-sum test, **Fig 3A**), confirming the dosage sensitivity of the identified PTB genes. In addition to being referenced with the genome background, direct comparisons among the PTB, term and GoNL groups also confirmed the significant increase in CNV intolerance in the PTB group (P = 0.05 between PTB and term groups, and P = 6.5e-3 between PTB and GoNL groups, Wilcoxon rank-sum test). Dividing CNV events into deletion and duplication events, the original study further quantified gene intolerance to deletions or duplications, and found that across the human genome, deletion intolerance is significantly correlated with duplication intolerance[26]. However, for this set of PTB genes, this was not the case. These PTB genes showed remarkable increased deletion intolerance (P = 2.8e-3, Wilcoxon rank-sum test, **Fig 3A**), but not duplication intolerance (P = 0.11, Wilcoxon rank-sum test, **Fig 3A**). This observation suggests that the aforementioned CNV intolerance should be specifically explained by their deletion intolerance. Again, the same signal was not observed in term infant genes or GoNL genes (both deletion and duplication intolerances), confirming the deleterious effects by specifically ablating the identified PTB genes.

To further characterize the phenotypic consequences of deleting the identified PTB genes, we examined their corresponding mouse mutants. In the ongoing effort of International Mouse Phenotyping Consortium to generate knockout mouse lines, 410 essential genes have been identified in the first 1,751 unique gene knockouts, whose homozygous deletions resulted in embryonic lethality, together with 198 and 1143 genes for subviable and viable phenotypes, respectively[27]. By mapping the mouse genes onto human orthologs, we examined the percentage of lethal, subviable and viable genes in the gene sets identified from PTB, term and GoNL individuals. Referenced with the term infant gene set, we found that the PTB infant set was highly enriched for genes whose mouse orthologs are considered essential (*i*.*e*. generating embryonic lethal phenotypes in their mutants, P = 7.7e-3, Fisher’s exact test, **Fig 3B**), but the GoNL set followed the same distribution as observed from the term set (P>0.9, Fisher’s exact test, **Fig 3B**). These data inquiries demonstrate the developmental significance of the identified genes affected by *de novo* mutations in PTB genomes, which in turn informs their deletion intolerance observed from our human genome analysis (**Fig 3A**).

### The role of abnormal fetal brain development in PTB

To gain mechanistic insights, we further examined specific phenotypes associated with the identified genes based on their mammalian phenotype ontology annotations[28]. We found that, in addition to the lethal phenotypes (FDR = 1.6e-3, S5 Table), the 51 PTB infant genes showed an enrichment for those causal for abnormal nervous system development (false discovery rate, FDR = 0.018, see Methods and Materials, S5 Table) and abnormal neuron physiology (FDR = 0.03, see Methods and Materials, S5 Table), whereas the enrichment was absent in the genes identified from the term group of infants as well as from the GoNL cohort (FDRs>0.2, S5 Table). These analyses indicate that abnormal nervous system development may contribute to PTB occurrence.

**Fig 4:**
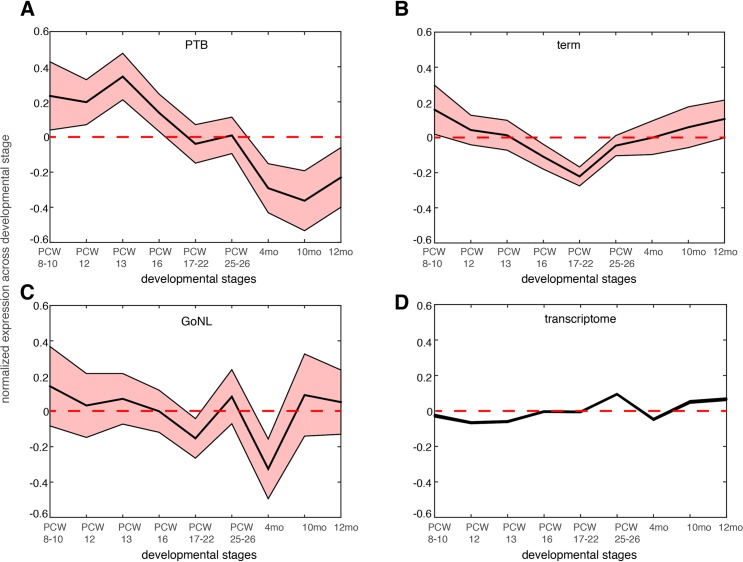
Gene expression dynamics during nine different neocortical developmental stages. The developmental stages include post-conceptual week (PCW) 8–10, PCW 8–12, PCW 13, PCW 16, PCW 17–22 and postnatal 4 months, 10 months and 12 months. The mean expression level together with the standard error of the mean (SEM) was plotted across the developmental stages for genes affected by de novo mutations identified in the PTB (panel **a**), term (panel **b**) and GoNL (panel **c**) cohorts. The same analysis was also performed on the entire transcriptome in each temporal epoch (panel **d,** the SEM is too small to be shown due to large sample size, *i*.*e*. overall 20,000 genes included). Gene expression values were normalized across different developmental stages.

We further reasoned that the 51 genes affected by the deleterious *de novo* mutations in the PTB infants should be preferentially expressed at prenatal development relative to postnatal stages. We examined the BrainSpan dataset[29], and studied the neocortical transcriptomic dynamics in post-conceptional week (PCW) 8–10, PCW 12, PCW 13, PCW 16, PCW 17–22, PCW 25–26 and postnatal 4 months, 10 months and 12 months[30]. Gene expression was normalized across these developmental temporal epochs. As shown in **Fig 4A**, we observed that the 51 genes identified from PTB infant groups showed the strongest expression propensity towards early fetal brain development (PCWs 8–10, 12, 13), and their expression decreased with the progress of the brain developmental stages, reaching the minimum at later postnatal stages. Testing on the 111 genes identified from the term group, on the 34 GoNL genes and on the entire transcriptome, such a pattern was not observed (**Fig 4B–4D**), thereby indicating specificity of these observations for contribution to PTB (**Fig 4A**).

The above analyses revealed a novel mechanism underlying PTB, which involved developmental abnormalities of the early fetal brain. If this is a common mechanism, *i*.*e*. not unique to this single study cohort, we would expect to observe similar findings in an independent study cohort. Because current and available genome-wide association studies (GWAS) were mostly focused on maternal genomes, and the GWAS signals themselves are hard to interpret (because of linkage disequilibrium), we examined copy number variants (CNVs) from our recent PTB study[31], where, 1,631 PTB (gestational age, 25^0^–29^6/7^ weeks) infant genomes were genotyped for CNVs (a subset of the newborns were diagnosed with bronchopulmonary dysplasia, a common pulmonary morbidity in PTB). The original study defined 131 broad large CNV regions (CNVRs, 74 deletions and 57 duplications) across all the PTB infants by collapsing SNPs of comparable statistical significance within a 1MB window[31].

**Fig 5:**
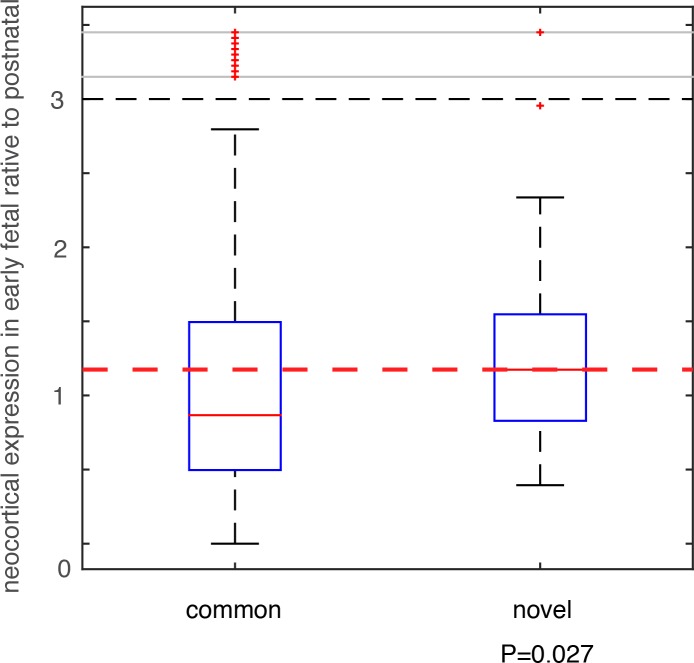
Testing on an independent PTB cohort. CNVs were identified in an independent PTB cohort, which were group into two classes based on their status as novel in PTB or as common in human population. Genes affected by these CNVs were identified, and their expression (β) in early fetal brain development stages (PCW 8–10, 12, and 13) relative to the postnatal stages (postnatal 4, 10 and 12 months) was compared between the two CNVs groups. Wilcoxon rank-sum test was used to determine statistical significance.

We compared these deletion and duplication CNVRs with those collected in the DGV database (Database of Genomic Variants), which is thus far the most comprehensive database collecting and curating known structural variants in the human genome[32]. All CNVs in DGV (392,583 CNV regions) were from non-diseased individuals. Among the 74 deletion CNVRs in this additional PTB cohort, 64 had been included in DGV, and 10 were novel (see Methods and Materials, and S6 Table). For the 57 duplication CNVRs, 56 were included in DGV, with only one novel (involving only one gene *MYO9A*, S6 Table). Therefore, we focused on the 10 PTB-specific novel deletion events. For genes affected by these deletion events, we analyzed their brain expression as we described above for the *de novo* mutations. Specifically, for each gene, we calculated the fold change (β) of its mean expression in PCWs 8–10, 12, 13 (early fetal development) relative to its mean expression in postnatal months 4, 10 and 12 (**Fig 4**). Therefore, a larger β value indicates a stronger expression propensity towards early fetal brain development. For genes affected by the 10 PTB-specific novel deletion events, we consistently observed a substantial increase in their expression propensity towards early fetal brain development (β>1, P = 0.027, Wilcoxon rank-sum test, **Fig 5**), whereas genes affected by the 64 common deletion events (shared with the DGV control cohort) exhibited a strong bias towards postnatal expression (β**<**1, **Fig 5**). Such an observed contrast between the PTB-specific and non-specific CNVs provides additional evidence for our observation implicating *de novo* mutations, and indicating that abnormalities in early fetal brain development may be causally associated with PTB.

## Discussion

To date, genetic studies of PTB have been primarily based on association studies for candidate genes or by whole genome scans with few at-risk loci robustly identified[15, 33]. Regardless of targeting the maternal or fetal genomes, such association studies, under the assumption of “common disease—common variant”, have been designed for common variants in human populations[34]. However, considering the high mortality of PTB newborns, PTB might be expected to be under severe natural selection pressure during human evolution. Thus, PTB-associated mutations would be expected to have substantially reduced allele frequencies, and therefore rare mutations would be more likely to play an etiologic role in PTB occurrence. This notion has been supported by our recent work on bronchopulmonary dysplasia, a common morbidity of PTB, where analyses of rare variants have identified a number of gene candidates for this disease[35], but association studies have not yet identified significant hits. Particularly for *de novo* mutations, given a lack of long-term selection force during human evolution, their effects are usually more deleterious than more neutral variants widely seen in human population[36].

This work the first study to systematically investigate *de novo* mutations in PTB infant genomes. We observed a significantly increased *de novo* mutation burden in PTB newborns. Our genome analyses further revealed that the affected genes by these PTB-associated *de novo* mutations are highly intolerant to genomic deletions, and their mouse mutants are embryonically lethal. Our analyses suggest the function of these PTB genes in early fetal brain development. These observations indicate a previously uncharacterized molecular etiology in PTB, where, independent of maternal genetics, spontaneous mutations in fetal genomes alone may contribute to PTB risk by perturbing the early brain development program in fetuses. These findings confirmed a significant genetic component in PTB. This work will potentially advance our understanding of PTB in many aspects.

On the maternal side, genes involved in immunity and inflammation have been associated with PTB[3, 4]. Such an association may also be biologically connected with our findings on the perturbation of the fetal nervous system in PTB. Recently, it has been shown that maternal inflammation disrupts fetal neurodevelopment[37, 38], and could even promote neuropsychiatric disorders of fetuses[39, 40]. Therefore, it is possible that fetal brain developmental abnormalities, at least in some cases, is a downstream event of maternal inflammation, both contributing to PTB as components in an integrated system. In our previous epidemiologic work, we observed that central nervous system malformations (malformed development predating the delivery event) were 10-fold more prevalent in infants born <31 weeks compared to infants born >36 weeks[41], and long-term neuropsychiatric impairment has been known as a common outcome of PTB[42, 43]. However, as revealed by this study, the presence of *de novo* mutations that affect fetal brain development argues that at least in some PTB cases, abnormal brain development in fetuses should precede PTB. In this scenario, it is anticipated that the abnormal brain developmental status of fetuses should be monitored by the fetal-maternal communication system, which could initiate the PTB process. Therefore, the role of abnormal fetal brain development in PTB is still in the exploratory phase, and future research is thus warranted to investigate the mechanistic links between PTB and fetal brain developmental abnormalities.

The contribution to PTB of infants paternally derived remains controversial. Epidemiological studies have suggested a weak impact from paternal genes[13, 44]; however, paternal age has been positively associated with PTB risk[22, 45, 46]. Interestingly, the number of *de novo* mutations is strongly scaled by paternal age[19–21] and the vast majority of *de novo* mutations are on paternal alleles (~80% in this study)[20]. Our study has thus suggested a potential path of paternal contribution to PTB.

To study the functional role of these *de novo* mutations in PTB genomes, we performed systematic functional genomic analyses. We showed that the affected genes are highly intolerant to genomic deletions, which is consistent with their essential role during embryonic development in their respective mouse models. This suggests that abnormal fetal development likely contributes to PTB. Specifically, our analyses further demonstrate the role of fetal brain development in PTB, we studied an independent PTB cohort, and tested our observations from *de novo* mutations on CNVs. This finding was important because it confirmed fetal brain development in PTB as a potentially common mechanism, not specific for a particular sample set, nor for a particular mutation type. The scenario is comparable with autism spectrum disorders, where a few specific biological pathways have been consistently identified regardless of diverse patient cohorts or mutation types analyzed[47]. Therefore, despite the seemingly heterogeneous mutations in PTB, these mutations may in fact converge onto a common set of biological pathways. Therefore, future integrative analysis is warranted to decipher the genetic etiology of PTB.

In our study, we compared the *de novo* mutation burden between PTB and term births following the conventional PTB definition of less than 37 gestational weeks. We also examined *de novo* mutation load in different gestational age ranges, and observed the strongest signal from the gestational age 32–37 weeks, and the enrichment was attenuated when considering PTB infants with gestational age less than 32 weeks. We reasoned that it could be due to insufficient sample size because most PTB infants (72.6%) were in the range of 32–37 weeks (**Fig 1**). However, an alternative plausible scenario could be attributed to a reduced survival (to detection) of the extreme PTB infants, who presumably harbor a high level of *de novo* mutation load. Because we studied live births, a further increase in *de novo* mutation burden in extreme PTB infants might not have been captured. In addition, our study compared PTB and term groups; however, the associated PTB subtype (*i*.*e*., spontaneous or medically indicated) for each trio was not available. Therefore, our observations are generalizable to PTB overall with its attendant underlying heterogeneity, and generalization to specific PTB subtypes requires further investigation.

In this study, we only studied non-synonymous mutations, which accounted for a small fraction of the spontaneous mutations (considering, on average, only one exonic *de novo* mutations among ~50 *de novo* mutations per genome[19–21, 23]). Given the significant impact of non-coding mutations in complex human diseases[48], it is expected that the role of fetal *de novo* mutations in PTB may very well be substantial. Taken together, our study reveals a novel etiology in PTB and thus opens a new avenue for future PTB research.

## Methods and materials

### The dataset of de novo mutations

A recent paper performed whole-genome sequencing (~60X) on whole blood samples from 816 parent-offspring trios and identified genomic signatures associated with *de novo* mutations[20]. These participating families are in general good health, and specifically the neonates had no known chromosomal abnormalities, genetic diseases and gross structural anatomic anomalies (see detailed description in the original publication). We obtained the *de novo* mutation dataset from this published study, together with information about gestational, paternal and maternal age for each offspring in these families. 292 newborns were preterm (gestational week <37 weeks), and the remaining 524 were born at term (gestational week ≥37 weeks). The high quality of *de novo* mutations was established in the original study. In total 36,441 autosomal *de novo* mutations were identified, including 648 small insertions/deletions (indels). We performed logistic regression and used paternal age, maternal age and the number of *de novo* mutations per fetal genome to predict the likelihood of being PTB. The three predictor variables were z-score normalized. For external control purposes, we also retrieved another *de novo* mutation dataset from whole-genome sequencing of 250 Dutch parent-offspring trio families, where the offspring had no known diseases[19] (GoNL: http://www.nlgenome.nl).

### Genomic resources

DNA replication timing was obtained from a previously published study, and we considered the data in human embryonic stem cells (the BG01 cell line)[24]. For each *de novo* mutation, we obtained the genomic coordinates (hg19) of a sequence window spanning 1kb centered at the mutation locus. The average DNA replication timing across the sequence window was then used to define the sequence context of each de novo mutation in fetal genomes. We annotated all the *de novo* mutations using wANNOVAR[49]. The annotation was based on the reference human genome build hg19, and the RefSeq gene definition. This annotation system automatically identified mutational consequences for each mutation (e.g. intronic, non-synonymous, frameshift, etc.) as well as the deleteriousness of the mutations by implementing several other prediction algorithms. We considered the mutational deleteriousness score defined by CADD (Combined Annotation Dependent Deletion), whose accuracy has outperformed many other methods[25]. We considered deleterious mutations if the nonsynonymous mutations were assigned with CADD phred-score greater than 20, meaning that these mutations were among the top 1% most deleterious mutations across the human genome. Therefore, this practice was conservative. We automatically consider frameshift indels deleterious.

CNV intolerance scores were obtained from a recent publication[26], where the intolerance to deletions, duplications and CNVs (combined set of deletions and duplications) were separately compared in this study. The scores were quantified for each gene, and a higher intolerance score indicates stronger selective pressure against CNVs on a particular gene. The mouse essential genes, whose homozygous knockouts displayed lethal phenotypes, were retrieved from a recent study[27], and the mouse genes were mapped onto their human orthologs based on Ensembl annotations (Ensembl Genes 86, GRCm38.p4). A mouse phenotypic enrichment test was performed based on the resources from Mouse Genome Informatics[28], and the implementation of the test was based on EnrichR (http://amp.pharm.mssm.edu/Enrichr/, as of October, 2016). All *p* values in these analyses have been adjusted for multiple hypothesis tests. We examined the BrainSpan dataset for gene expression dynamics across neocortical developmental stages[29, 30]. We normalized gene expression across different developmental stages, which allowed us to identify gene expression propensities towards particular temporal epochs. The original data (unnormalized) were used to determine the fold change (β) of gene expression in early fetal brain development relative to postal stages.

We obtained copy number variants (CNVs) data from our previous study, where CNVs were identified in 1,631 PTB infants (gestational age, 25^0^−29^6/7^ weeks)[31]. The original study defined 131 large CNV regions (CNVRs, 74 deletions and 57 duplications) across all the PTB infants by collapsing SNPs of comparable statistical significance within a 1MB window. To identify CNV regions commonly seen in human population, we retrieved CNVs collected in DGV (Database of Genomic Variants, genome build hg19, http://dgv.tcag.ca/dgv/app/home)), where 392,583 CNVs (as of October, 2016) in non-diseased individuals have been collected in the database[32]. We considered deletion events in the database for CNVs with the mutation type annotated as “deletion” or “loss”, and duplication events as “duplication” or “gain”. In our comparison, we consider a deletion event that is shared with DGV, if at least 80% of the deletion region can also be found in the DGV deletion collection, or we consider it novel. The practice was also applied to defining the novelty of duplication regions. Bedtools was used for this comparison (http://bedtools.readthedocs.io/en/latest/).

## Supporting information

S1 Table The final *de novo* mutation calls in each individual. Mutation calls were split into PTB and non-PTB individuals (two separate data sheets), and their respective subject ID and gestational age (in weeks) are also indicated.
(XLSX)

S2 Table Information about the samples in this study. Paternal, maternal and gestational age (in days and weeks) are provided.
(XLSX)

S3 Table *De novo* mutation count for each newborn genome. The corresponding gestational age and the results from logistic regression analysis (in the second data sheet) are also provided.
(XLSX)

S4 Table Mutational consequences of the *de novo* mutations. Non-synonymous *de novo* mutations were identified, and the mutational consequences were computed using CADD phred-scores. The data for PTB and non-PTB individuals were shown in two different datasheets.
(XLSX)

S5 Table Enrichment test for mouse mutant phenotypes. The test results for PTB, non-PTB and GoNL cohorts were separately shown in three different datasheets.
(XLSX)

S6 Table The lists of novel or common deletion and duplication events (two datasheets). (XLSX)
